# Impact of obesity on clinical outcomes and the treatment effect of catheter ablation in atrial fibrillation: insights from CABANA

**DOI:** 10.1093/europace/euag293

**Published:** 2026-10-29

**Authors:** Jonas Erzeel, Sebastiaan Dhont, Marnicq van Es, Michiel De Wever, Evelyne Meekers, Hugo Van Herendael, Dieter Nuyens, Maximo Rivero-Ayerza, Laurent Pison, Wilfried Mullens, Wai Hong Wilson Tang, Pieter Martens

**Affiliations:** Department of Cardiology, Ziekenhuis Oost-Limburg A.V., Genk, Belgium; UHasselt, Faculty of Medicine and Life Sciences, Agoralaan, 3590 Diepenbeek, Belgium / LCRC(-MHU); Department of Cardiology, Ziekenhuis Oost-Limburg A.V., Genk, Belgium; UHasselt, Faculty of Medicine and Life Sciences, Agoralaan, 3590 Diepenbeek, Belgium / LCRC(-MHU); Department of Cardiology, Ziekenhuis Oost-Limburg A.V., Genk, Belgium; UHasselt, Faculty of Medicine and Life Sciences, Agoralaan, 3590 Diepenbeek, Belgium / LCRC(-MHU); Department of Cardiology, Ziekenhuis Oost-Limburg A.V., Genk, Belgium; UHasselt, Faculty of Medicine and Life Sciences, Agoralaan, 3590 Diepenbeek, Belgium / LCRC(-MHU); Department of Cardiology, Ziekenhuis Oost-Limburg A.V., Genk, Belgium; UHasselt, Faculty of Medicine and Life Sciences, Agoralaan, 3590 Diepenbeek, Belgium / LCRC(-MHU); Department of Cardiology, Ziekenhuis Oost-Limburg A.V., Genk, Belgium; Department of Cardiology, Ziekenhuis Oost-Limburg A.V., Genk, Belgium; Department of Cardiology, Ziekenhuis Oost-Limburg A.V., Genk, Belgium; Department of Cardiology, Ziekenhuis Oost-Limburg A.V., Genk, Belgium; UHasselt, Faculty of Medicine and Life Sciences, Agoralaan, 3590 Diepenbeek, Belgium / LCRC(-MHU); Department of Cardiology, Ziekenhuis Oost-Limburg A.V., Genk, Belgium; UHasselt, Faculty of Medicine and Life Sciences, Agoralaan, 3590 Diepenbeek, Belgium / LCRC(-MHU); Department of Cardiovascular Medicine, Heart, Vascular and Thoracic Institute, Cleveland Clinic, 9500 Euclid Avenue, Desk J3-4, Cleveland, OH 44195, USA; Department of Cardiology, Ziekenhuis Oost-Limburg A.V., Genk, Belgium; UHasselt, Faculty of Medicine and Life Sciences, Agoralaan, 3590 Diepenbeek, Belgium / LCRC(-MHU)

**Keywords:** Obesity, Atrial fibrillation, Comorbidities, Catheter ablation, Randomized controlled trials

## Abstract

**Aims:**

Obesity and atrial fibrillation (AF) are global epidemics, often co-existing and compounding cardiovascular risk. While catheter ablation has emerged as an effective strategy for rhythm control in symptomatic AF, it remains unclear whether obesity modifies its benefit. This *post hoc* analysis of the CABANA trial investigates the influence of baseline obesity on clinical outcomes after catheter ablation vs. drug therapy in AF patients.

**Methods and results:**

A total of 2170 CABANA participants with available body mass index (BMI) were included. Obesity was defined by BMI ≥ 30 kg/m^2^, and Class II obesity by BMI ≥ 35 kg/m^2^. Adverse outcomes, AF recurrence, and functional status were analysed using Cox, mixed-effects, and generalized estimating equation models, assessing treatment-by-obesity interactions. Obese patients exhibited higher comorbidity burden, worse baseline functional status, and more persistent AF compared to non-obese counterparts. Higher BMI was associated with increased cardiovascular hospitalization and a higher New York Heart Association functional class. Catheter ablation significantly reduced the risk of AF recurrence and improved symptom burden and functional capacity, irrespective of obesity status. No significant interaction was found between BMI and treatment effect on the primary composite endpoint of cardiovascular hospitalization or all-cause mortality. However, patients with Class II obesity or higher had worse absolute outcomes despite similar relative benefits from ablation.

**Conclusion:**

While obesity is associated with worse baseline status and outcomes, it was not shown to significantly alter the relative effectiveness of AF ablation compared to drug therapy. These findings support a multifaceted approach integrating rhythm control with upstream interventions targeting obesity-related atrial remodelling and comorbidity management.

## Graphical Abstract

**Graphical Abstract:**
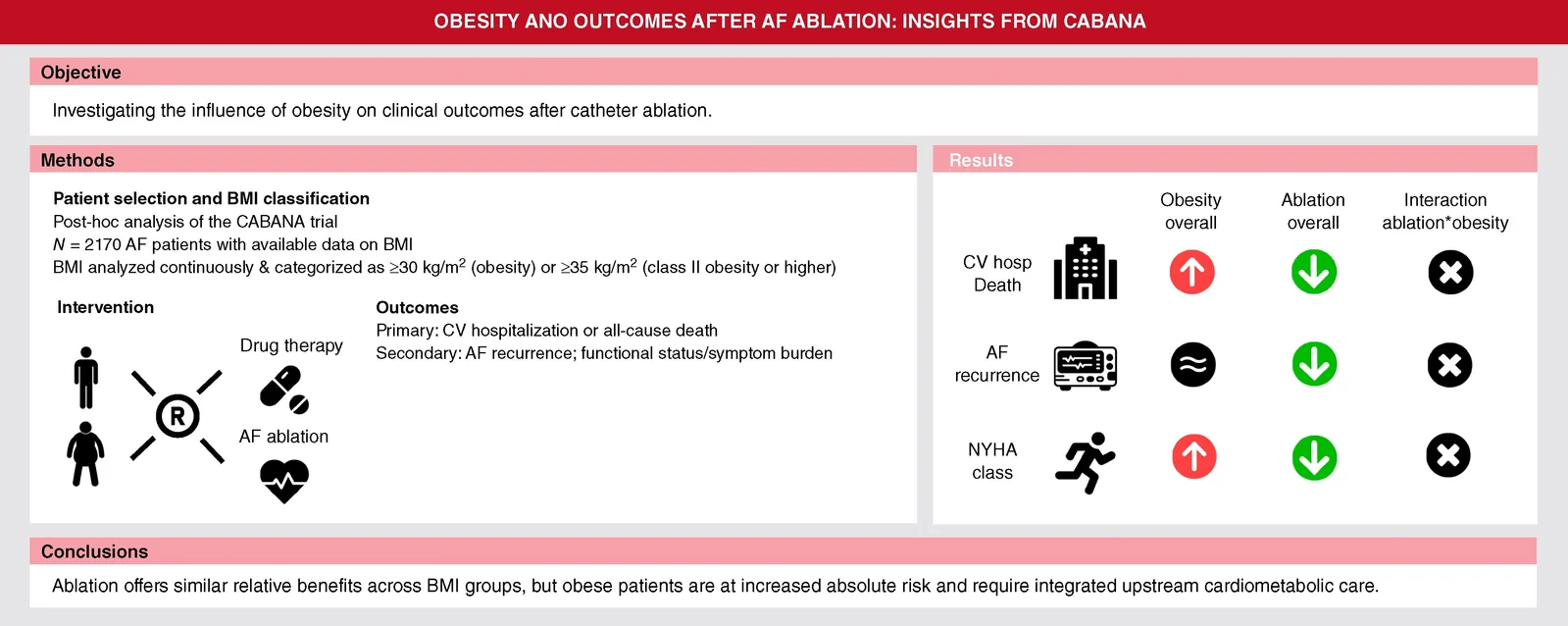

What’s New

- This exploratory *post hoc* analysis of 2170 CABANA participants assessed whether baseline obesity modifies the effects of catheter ablation versus drug therapy for atrial fibrillation.
- Higher body mass index (BMI) was associated with increased cardiovascular hospitalization and worse functional status; patients with BMI ≥ 35 kg/m² also had a higher risk of cardiovascular hospitalization or all-cause death.
- No significant treatment-by-BMI interactions were detected for clinical outcomes, atrial fibrillation recurrence, functional status, or symptom severity, providing no evidence that obesity attenuated the relative benefits of ablation.
- Despite improvements with ablation, patients with obesity had greater residual functional impairment, supporting comprehensive management addressing obesity and associated comorbidities alongside rhythm control.

## Introduction

Atrial fibrillation (AF) is the most common sustained cardiac arrhythmia worldwide, affecting over 52 million people and contributing substantially to morbidity and healthcare burden, with its global prevalence and associated disease burden projected to increase markedly over the coming decades.^1–3^ Catheter ablation has become a cornerstone in rhythm control for patients with symptomatic AF.^4^ While ablation is more effective than antiarrhythmic drugs in maintaining sinus rhythm and improving quality of life, its impact on long-term clinical outcomes, such as mortality, remains debated.^5^ In the CABANA trial (Catheter Ablation vs. Antiarrhythmic Drug Therapy for Atrial Fibrillation), no significant difference was found in the composite primary endpoint (death, disabling stroke, serious bleeding, or cardiac arrest) between ablation and drug therapy groups.^6^ Nevertheless, ablation was associated with fewer recurrences of AF and reduced cardiovascular (CV) hospitalizations, highlighting its role in improving rhythm control and reducing symptom burden.

In parallel, obesity has reached epidemic proportions globally, with over one billion individuals affected.^7^ Obesity [body mass index (BMI) ≥ 30 kg/m^2^] is a well-established independent risk factor for both incident AF and its progression to more persistent forms.^8^ Mechanistically, obesity contributes to atrial remodelling through multiple interrelated pathways, including left atrial dilation, increased epicardial adipose tissue, systemic and local inflammation, and atrial fibrosis.^9,10^ This disrupts electrical conduction and promotes structural remodelling, creating a substrate conducive to AF. Moreover, autonomic dysfunction and activation of neurohormonal pathways, such as the renin–angiotensin–aldosterone system, further exacerbate arrhythmogenesis.^11^

Given these mechanisms, it is plausible that obesity may influence the efficacy of catheter ablation. Several observational studies and meta-analyses have reported higher rates of AF recurrence, increased procedural complexity, and worse functional outcomes in obese patients undergoing AF ablation.^12–14^ However, the observational nature of such analyses does not allow for conclusions about whether obese patients are simply at increased risk of AF recurrence and worse functional outcome in general, or if obesity is associated with a diminished treatment effect of ablation. Conversely, weight reduction has been associated with improved ablation outcomes and reduced AF burden.^15–18^ To clarify this relationship, we conducted a *post hoc* analysis of the CABANA trial, investigating whether baseline obesity modifies the treatment effect of catheter ablation compared to drug therapy in a randomized population.

## Methods

### Data availability

The CABANA trial data were obtained through the publicly available National Heart, Lung, and Blood Institute BioLINCC data repository, and this analysis was approved by the Cleveland Clinic Institutional Review Board as an exempt study. The authors have full access to all study data, take responsibility for its integrity and the accuracy of the analyses, and may agree to provide aggregate data upon reasonable request and approval by the Institutional Review Board.

### Trial design and patient population

This study is a *post hoc* analysis of the CABANA randomized controlled trial (NCT00911508), which evaluated catheter ablation vs. drug therapy for AF, for which the design and results have previously been published.^6^ The present BMI/obesity analyses, including treatment-effect modification analyses, were exploratory and were not pre-specified in the original CABANA statistical analysis plan. The trial enrolled patients aged ≥ 65 years or those aged < 65 years with at least one stroke risk factor (e.g. hypertension, heart failure, prior stroke or transient ischaemic attack, or diabetes). Eligible participants had experienced at least two symptomatic episodes of paroxysmal AF or a single episode of persistent AF within the 6 months prior to randomization. Participants were randomized in a 1:1 ratio to receive either catheter ablation or drug therapy (including rate or rhythm control strategies). Clinical endpoints were assessed throughout a standard follow-up period up to 60 months, with scheduled visits approximately every 3 months during the first 6 months and every 6 months thereafter. All participants provided written informed consent, and an institutional review board or ethics committee approval was obtained at each participating centre. The CABANA trial adhered to the Declaration of Helsinki and was conducted in accordance with STROBE guidelines for observational research derived from randomized trials.

### Intervention

In the CABANA trial, participants received either catheter ablation or drug therapy as initial treatment for AF, with randomization stratified by clinical site. The primary goal of the ablation strategy was elimination of AF through pulmonary vein isolation. The specific ablation approach, energy source, and treatment of any additional lesions beyond pulmonary vein isolation were left to the discretion of the treating electrophysiologist. Radiofrequency energy was the predominant ablation modality used during the CABANA trial. Patients assigned to the drug therapy group were initially managed with rate control strategies, aiming to achieve a resting heart rate of <80 beats per minute and an exercise heart rate of <110 beats per minute, assessed via 6-minute walk testing or Holter monitoring. If rate control targets were not met, or symptoms persisted, rhythm control could be initiated using antiarrhythmic drugs, selected according to individual clinical profiles. Electrical cardioversion was permitted as needed. Ablation of the atrioventricular node was allowed in cases of refractory rate control despite multiple pharmacological attempts. Both treatment strategies were implemented in accordance with clinical judgement, reflecting real-world practice variability. Randomization and treatment delivery were not influenced by BMI or obesity status.

### Obesity status

For the current analysis, we evaluated the influence of baseline obesity on the treatment response of AF ablation. Obesity was defined using BMI, which was recorded at the time of trial enrolment and was calculated as weight in kilograms divided by height in metres squared (kg/m^2^). Patients with missing BMI values were excluded from further analyses. Body mass index was analysed both as a continuous and categorical variable. In categorical analyses, obesity status was classified according to World Health Organization obesity definitions, using a BMI cut-off of 30 kg/m^2^ to distinguish obese and non-obese patients. In selected analyses, in which prior findings or continuous analysis suggested potential relevance, a higher cut-off of 35 kg/m^2^ was additionally applied to identify obesity Class II or greater, representing more severe adiposity.^19^ These categorical subgroupings were used in parallel to explore potential dose–response relationships and to evaluate whether the treatment effect varied by degree of obesity.

### Endpoints

For the current analysis, the primary endpoint was pre-specified as the composite of CV hospitalization or all-cause death during follow-up. Cardiovascular hospitalization included admissions for CV causes, such as arrhythmia, heart failure, ischaemic events, and other CV indications, according to the original CABANA trial endpoint definitions. The secondary endpoints included the individual components of the composite endpoint, AF recurrence during follow-up, and patient-reported functional status as measured by the New York Heart Association (NYHA) functional class and the Canadian Cardiovascular Society (CCS) Severity of Atrial Fibrillation scale. For AF recurrence, any electrocardiographic evidence of AF lasting >30 s during follow-up was considered sufficient, irrespective of antiarrhythmic drug use.

### Statistical analysis

Baseline characteristics and ablation procedural data were summarized as means with standard deviations, medians with interquartile ranges (25th–75th percentiles), or frequencies with percentages, as appropriate. Group comparisons were performed using independent sample *t*-tests, Mann–Whitney *U* tests, or χ^2^ tests, based on variable distribution and scale. The primary composite endpoint of CV hospitalization or all-cause death (or its separate components) was analysed using a Cox proportional hazards model, including both treatment group and BMI as fixed effects. Interaction terms between treatment assignment and obesity status were included to assess whether BMI modified the treatment effect. In continuous analysis, spline-based modelling was additionally employed to capture potential nonlinear associations between BMI and clinical outcomes, offering a flexible method to detect inflection points in the treatment effect across the BMI spectrum. Atrial fibrillation recurrence during follow-up was analysed as a binary outcome using a generalized linear mixed model with a logit link, incorporating a fixed treatment effect and random intercept. Treatment-effect modification was explored through adding the interaction terms of BMI or obesity status-by-treatment into the overall model, in addition to their main effects. Similarly, ordinal functional outcomes, such as NYHA class and CCS AF severity, were analysed using generalized estimating equation models with a cumulative logit link, including fixed effects for treatment, BMI, and their interaction. For NYHA class, additional analyses dichotomized the outcome (NYHA < 2 vs. ≥2) to reflect clinically significant functional impairment. All models followed the intention-to-treat principle. Analyses used all available observations at each time point, without excluding patients with partially missing follow-up data. For analyses evaluating the association between BMI (across treatment groups) and clinical outcomes, functional status, and AF recurrence, univariable models were supplemented with multivariable models adjusted for key baseline covariates, including age, AF type, hypertension, diabetes, obstructive sleep apnoea, coronary artery disease, and valvular heart disease. Hypothesis testing was two sided, with a significance threshold of *α* = 0.05. All statistical analyses were performed using R Statistical Software (R Foundation), version 4.4.0, and SPSS Statistics, version 29.0 (IBM Corp., Armonk, N.Y., USA).

## Results

### Baseline characteristics

The CABANA trial included a total of 2204 patients, of which 2170 had data on BMI available and were included in this analysis, with a median follow-up duration of 46.5 months. The baseline characteristics of these patients are shown in *Table 1* and Supplementary material online, *Table S1*, stratified by obesity status. Patients with and without available BMI data had similar baseline characteristics (see Supplementary material online, *Table S2*). Obese patients (BMI ≥ 30 kg/m^2^) comprised 1107 participants (51%), while 1063 (49%) were non-obese (BMI < 30 kg/m^2^) (see *Figure 1*). Obese patients were younger than their non-obese counterparts and had higher systolic blood pressure. Regarding AF phenotype, obese patients were less likely to present with paroxysmal AF and more likely to exhibit persistent or long-standing persistent AF. Symptom burden and functional status at baseline also differed modestly between groups. Obese patients were more often classified as NYHA class ≥ II and had higher CCS AF severity scores. Comorbidity burden was substantially higher among obese participants. Obese patients had significantly higher rates of hypertension, diabetes, sleep apnoea, and coronary artery disease. In contrast, valvular heart disease was less common in the obese cohort. Among patients randomized to catheter ablation, the procedure was carried out with no difference in rates between obese and non-obese patients (see Supplementary material online, *Table S3*). Similarly, there was no difference in catheter ablation–related adverse events according to obesity status (see Supplementary material online, *Table S4*).

**Figure 1:**
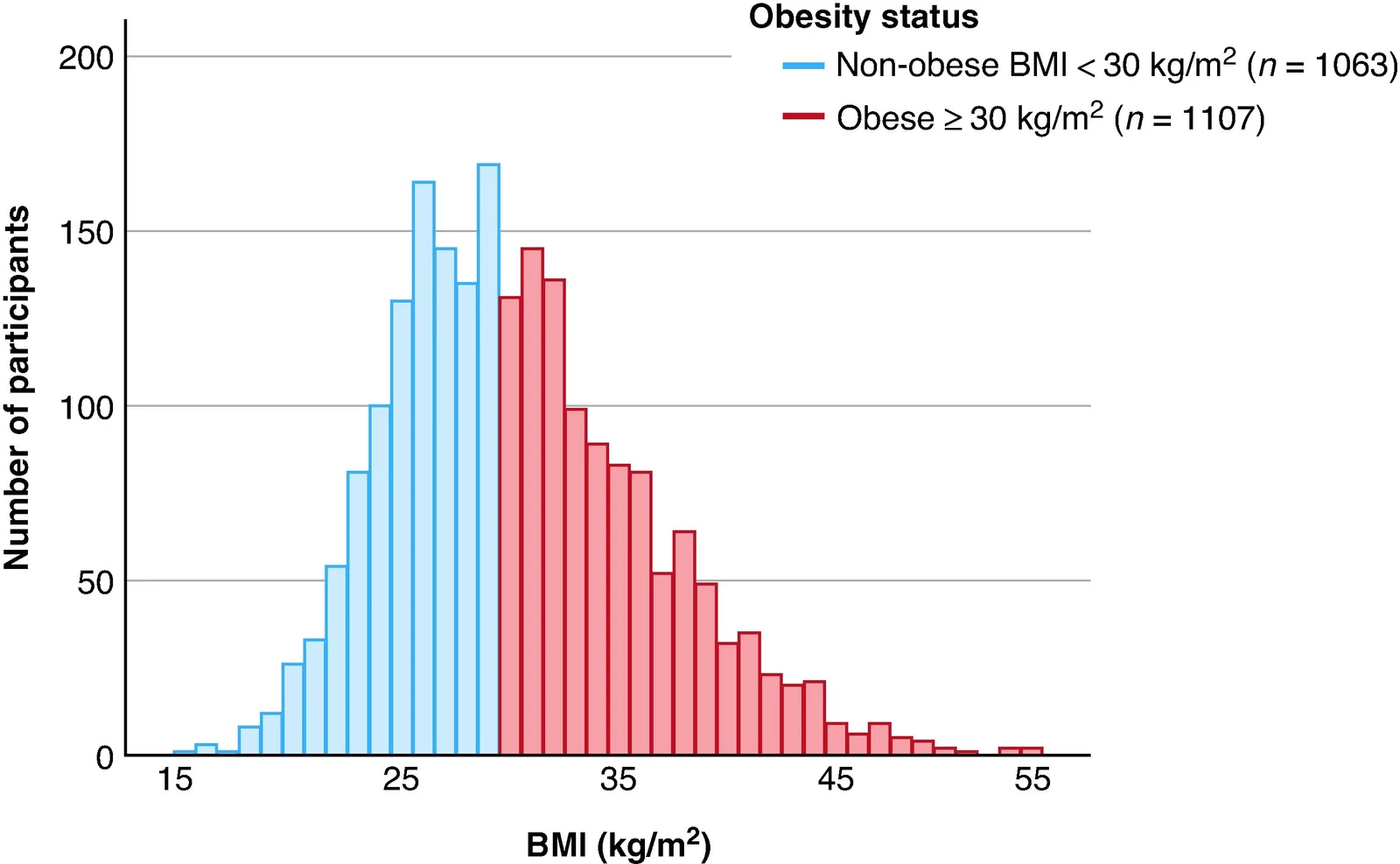
Histogram showing the distribution of BMI in obese (BMI ≥ 30 kg/m^2^) and non-obese (BMI < 30 kg/m^2^) patients at baseline. BMI, body mass index.

**Table 1:** Baseline characteristics of the patient population, stratified by obesity status

	Overall (*n* = 2170)	Non-obese (BMI < 30 kg/m^2^) (*n* = 1063)	Obese (BMI ≥ 30 kg/m^2^) (*n* = 1107)	*P* value
Clinical characteristics				
Age (years)	67.0 (62.0–72.0)	68.0 (63.0–73.0)	66.0 (60.0–70.0)	**<0**.**001**
Male	1370 (63.1)	675 (63.5)	695 (62.8)	0.729
BMI (kg/m^2^)	30.1 (26.5–34.6)	26.5 (24.4–28.3)	34.4 (32.0–38.3)	**<0**.**001**
Systolic blood pressure (mmHg)	129.0 (120.0–140.0)	127.5 (119.0–140.0)	130.0 (120.0–140.0)	**0**.**038**
Diastolic blood pressure (mmHg)	80.0 (70.0–84.0)	80.0 (70.0–84.0)	80.0 (70.0–84.0)	0.938
LVEF (%)	57.0 (50.0 -61.8)	57.8 (50.5–62.5)	56.0 (50.0–61.0)	**0**.**012**
AF type				
Paroxysmal	935 (43.1)	488 (45.9)	447 (40.4)	**0**.**014**
Persistent	1023 (47.1)	485 (45.6)	538 (48.6)	
Long-standing persistent	212 (9.8)	90 (8.5)	122 (11.0)	
NYHA functional class				
≤I	1387 (63.4)	744 (67.8)	673 (61.0)	**0**.**023**
II	586 (27.2)	263 (25.0)	323 (29.3)	
III	180 (8.4)	75 (7.1)	105 (9.5)	
IV	2 (<0.1)	1 (<0.1)	1 (<0.1)	
CCS AF severity score				
0	226 (10.4)	127 (11.9)	99 (8.9)	**0**.**036**
1	336 (15.5)	170 (16.0)	166 (15.0)	
2	696 (32.1)	350 (32.9)	346 (31.3)	
3	771 (35.5)	356 (33.5)	415 (37.5)	
4	141 (6.5)	60 (5.6)	81 (7.3)	
Comorbidities				
Hypertension	1750 (80.6)	775 (72.9)	975 (88.1)	**<0**.**001**
Diabetes	549 (25.3)	150 (14.1)	399 (36.0)	**<0**.**001**
Sleep apnoea	505 (23.3)	139 (13.1)	366 (33.1)	**<0**.**001**
Coronary artery disease	417 (19.2)	162 (15.2)	255 (23.0)	**<0**.**001**
Prior stroke	126 (5.8)	67 (6.3)	59 (5.3)	0.190
Thromboembolic events	89 (4.1)	45 (4.2)	44 (4.0)	0.917
Valvular disease	261 (12.0)	150 (14.1)	111 (10.0)	**0**.**002**
Family history of AF	248 (11.5)	115 (10.8)	133 (12.0)	0.210

Values are presented as *n* (%) or as median (25th–75th percentiles). *P* values in bold indicate statistical significance at *α* = 0.05.

BMI, body mass index; AF, atrial fibrillation; LVEF, left ventricular ejection fraction; NYHA, New York Heart Association; CCS, Canadian Cardiovascular Society.

### Clinical outcomes by body mass index and obesity status

*Table 2* illustrates the results of continuous and categorical analyses of the effect of BMI and obesity status on hard clinical endpoints across treatment groups. Analysed as a continuous variable, BMI was significantly associated with the composite endpoint of CV hospitalization or all-cause death and with CV hospitalization alone. Each 1 kg/m^2^ increase in BMI was associated with a 1.0% increase in hazard for both [hazard ratio (HR), 1.01; 95% confidence interval (CI), 1.00–1.02; *P* = 0.039 and HR, 1.01; 95% CI, 1.00–1.02; *P* = 0.038, respectively]. In contrast, no significant association was observed between BMI and death. Using a BMI threshold of ≥30 kg/m^2^ to compare obese and non-obese patients, no significant differences were found in the risk of the composite endpoint, CV hospitalization, or death alone. However, when a higher BMI cut-off of ≥35 kg/m^2^ was applied, patients with Class II obesity or higher demonstrated a significantly increased risk for both the composite endpoint (HR, 1.17; 95% CI, 1.02–1.34; *P* = 0.026) and CV hospitalization alone (HR, 1.16; 95% CI, 1.01–1.34; *P* = 0.033), though again, no significant association was observed with death. These associations remained consistent after multivariable adjustment (see Supplementary material online, *Table S5*).

**Table 2:** Effect of obesity status on clinical endpoints across treatment groups in patients with atrial fibrillation

Continuous BMI			
	Hazard ratio	95% CI	*P* value
Composite endpoint	1.01	1.00–1.02	**0.039**
CV hospitalization	1.01	1.00–1.02	**0**.**038**
All-cause death	1.01	0.98–1.04	0.744

Categorical analysis—cut-off BMI ≥ 30 kg/m^2^					
	Non-obese (*n* = 1063)	Obese (*n* = 1107)	Hazard ratio	95% CI	*P* value
Composite endpoint	531 (50.0)	601 (54.3)	1.07	0.95–1.20	0.254
CV hospitalization	505 (47.5)	579 (52.3)	1.09	0.96–1.22	0.179
All-cause death	63 (5.9)	57 (5.1)	0.850	0.59–1.22	0.373

Categorical analysis—cut-off BMI ≥ 35 kg/m^2^					
	Class 0–I obesity (*n* = 1574)	Class II–III obesity (*n* = 476)	Hazard ratio	95% CI	*P* value
Composite endpoint	856 (51.5)	276 (54.4)	1.17	1.02–1.34	**0**.**026**
CV hospitalization	821 (49.4)	263 (51.9)	1.16	1.01–1.34	**0**.**033**
All-cause death	89 (5.4)	31 (6.1)	1.18	0.78–1.78	0.427

Values are presented as *n* (%). Composite endpoint defined as CV hospitalization and/or all-cause death. *P* values in bold indicate statistical significance at *α* = 0.05.

BMI, body mass index; CV, cardiovascular; HR, hazard ratio; CI, confidence interval.

### Functional status by body mass index and obesity status

Across treatment groups, in continuous analysis, higher BMI was significantly associated with worse NYHA class during follow-up [odds ratio (OR) per 1 kg/m^2^ increase, 1.03; 95% CI, 1.02–1.04; *P* < 0.001]. In categorical analysis using a BMI threshold of ≥30 kg/m^2^, obese patients had 46% higher odds of being in worse NYHA class compared with non-obese patients (OR, 1.46; 95% CI, 1.24–1.73; *P* < 0.001). *Figure 2* displays the model-predicted percentages of patients in NYHA class ≥ II, pooled across all follow-up visits, illustrating the overall shift towards worse functional status in obese patients. Associations were similar in multivariable-adjusted models (adjusted OR per 1 kg/m^2^ increase, 1.03; 95% CI, 1.02–1.05; *P* < 0.001; adjusted OR BMI ≥ 30 kg/m^2^, 1.46; 95% CI, 1.20–1.78; *P* < 0.001).

**Figure 2:**
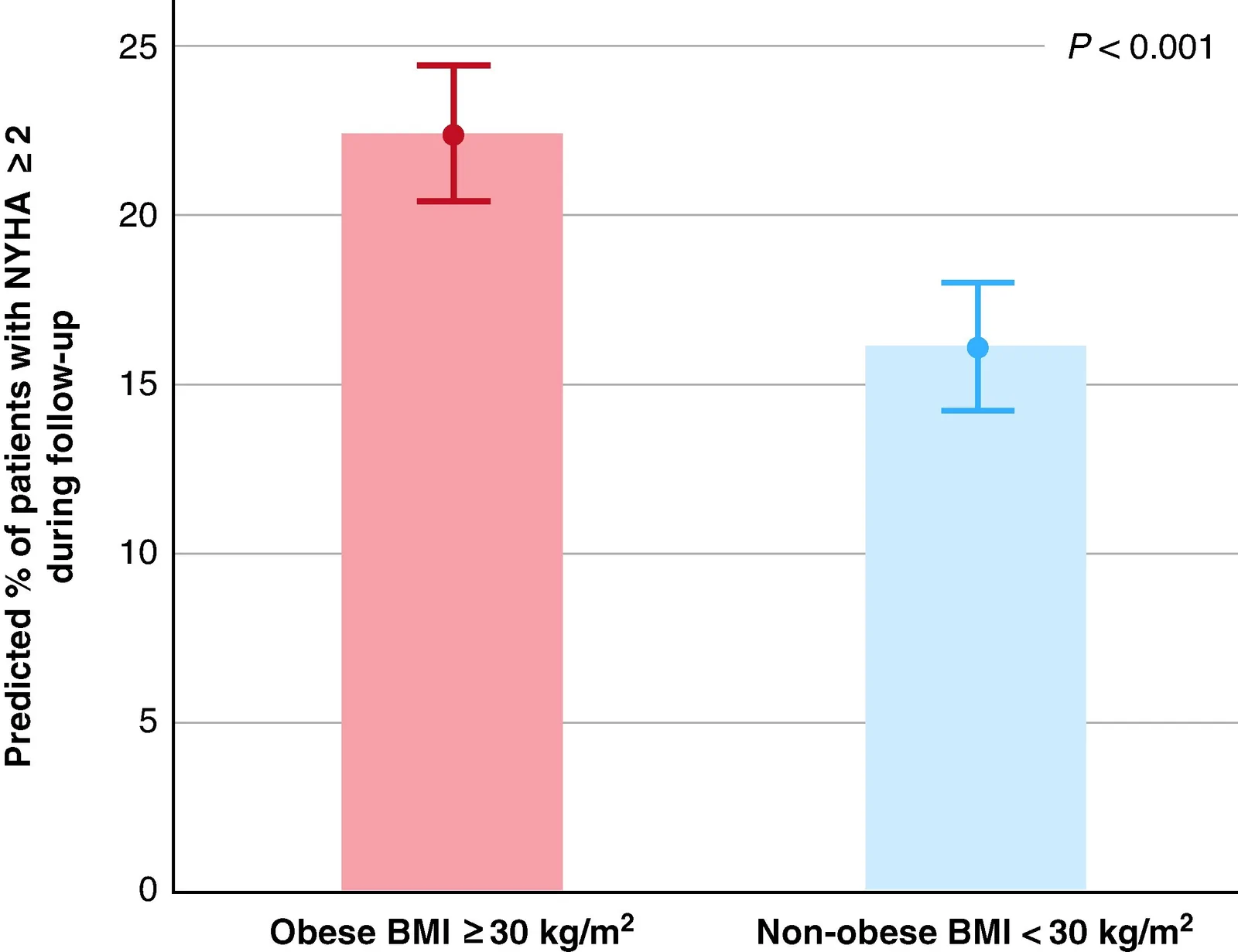
Generalized estimating equations model-predicted percentage of patients with NYHA class ≥ II during follow-up, stratified by obesity status (non-obese, BMI < 30 kg/m^2^; obese, BMI ≥ 30 kg/m^2^). Error bars represent 95% confidence intervals. BMI, body mass index; NYHA, New York Heart Association.

### Atrial fibrillation recurrence by body mass index and obesity status

Across treatment groups, in categorical analysis (BMI threshold ≥ 30 kg/m^2^), obese patients did not exhibit a significant difference in AF recurrence (OR, 1.03; 95% CI, 0.88–1.20; *P* = 0.745). Similarly, when BMI was analysed as a continuous variable, no significant association with recurrence was observed (OR per 1 kg/m^2^ increase, 1.00; 95% CI, 0.99–1.01; *P* = 0.921). Associations were similar in multivariable-adjusted models (adjusted OR per 1 kg/m^2^ increase, 1.00; 95% CI, 0.98–1.01; *P* = 0.403; adjusted OR BMI ≥ 30 kg/m^2^, 1.05; 95% CI, 0.88–1.25; *P* = 0.597).

### Impact of obesity on the effects of atrial fibrillation ablation on clinical outcomes

*Figure 3* visualizes the treatment effect of AF ablation on the composite endpoint of CV hospitalization or death and CV hospitalization alone for both the overall group and stratified by obesity status. In the overall population, ablation was associated with a significant reduction in the risk of the composite endpoint compared to drug therapy (HR, 0.88; 95% CI, 0.78–0.99; *P* = 0.032). Obesity status did not seem to significantly modify the treatment effect of AF ablation when using BMI cut-offs of ≥30 and ≥35 kg/m^2^, as shown by non-significant interaction terms (*P* for interaction = 0.494 and 0.268, respectively; see Supplementary material online, *Table S6*). Likewise, for CV hospitalization alone, no significant interactions were observed between treatment and obesity status (*P* for interaction = 0.609 and 0.316, respectively).

**Figure 3:**
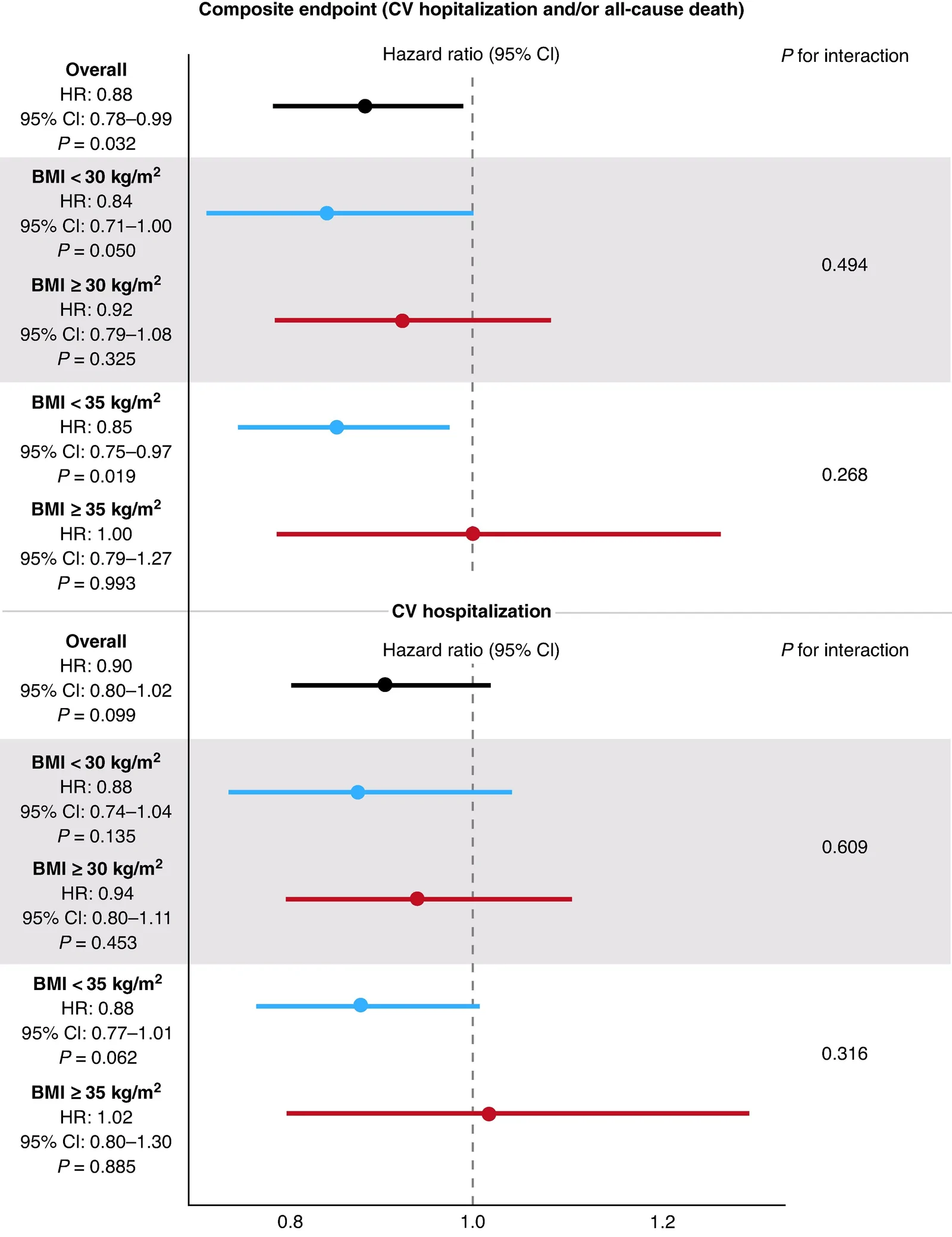
Forest plots displaying Cox proportional hazard ratios and 95% confidence intervals for the composite endpoint (CV hospitalizations or all-cause death) and CV hospitalization alone, comparing atrial fibrillation ablation to drug therapy across obesity subgroups. CI, confidence interval; CV, cardiovascular.

Similar to the categorical analysis of BMI, when BMI was analysed as a continuous variable, there was no significant interaction between BMI and the treatment effect of AF ablation on the composite endpoint or CV hospitalization alone (*P* for interaction = 0.716 and 0.706, respectively). *Figure 4* visualizes the treatment effect of AF ablation by BMI for the composite endpoint as a restricted cubic spline. For the individual endpoint of all-cause death, no statistically significant treatment effects were observed in either obesity subgroup across both thresholds, and the interaction terms were also not significant both in categorical and continuous analyses.

**Figure 4:**
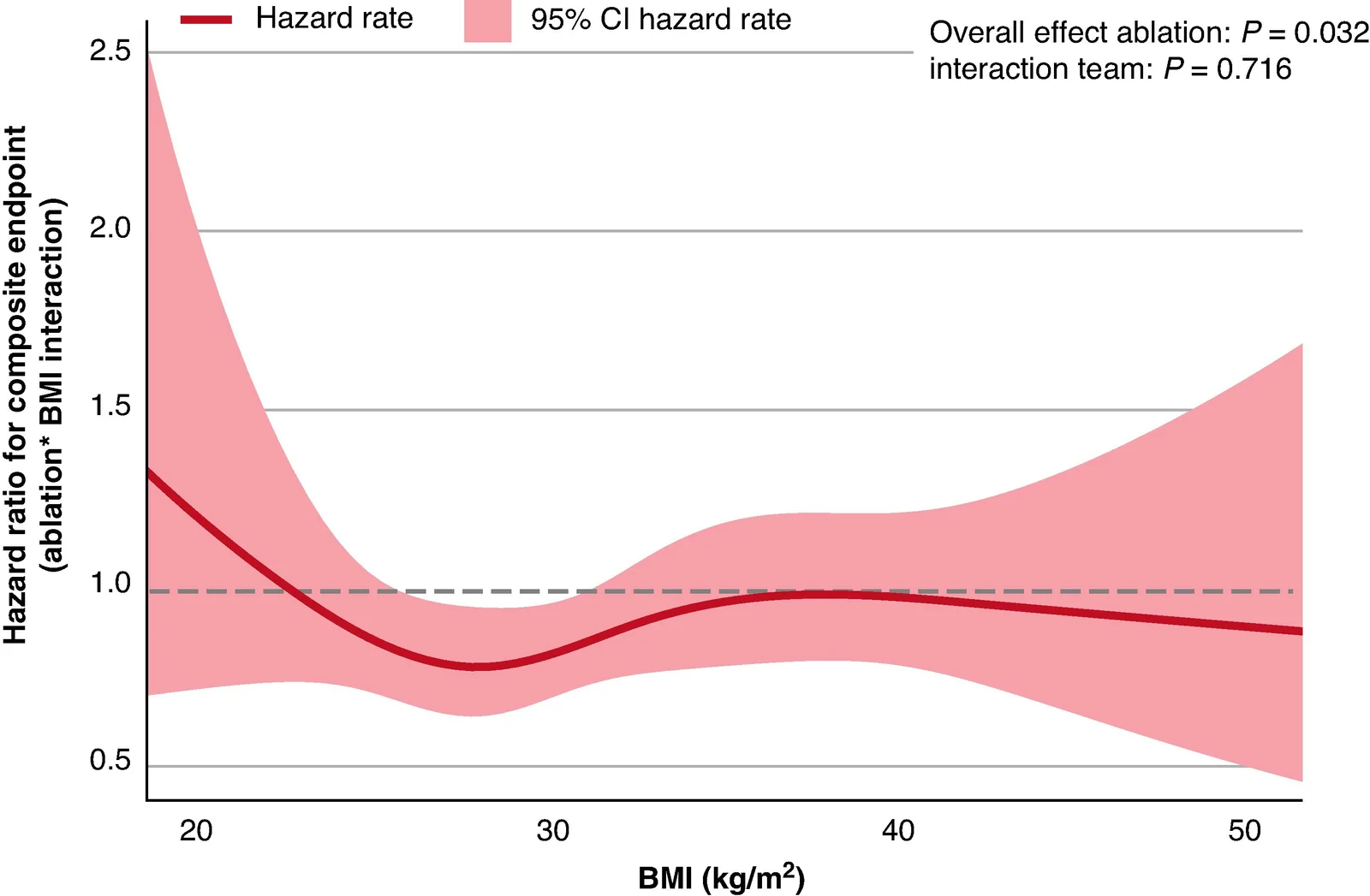
Restricted cubic spline analysis illustrating the continuous interaction between BMI and the treatment effect of atrial fibrillation ablation on the hazard ratio for the composite endpoint. HR < 1.0 favours ablation; HR > 1.0 favours drug therapy. Shaded region represents 95% CI. BMI, body mass index; CI, confidence interval, HR, hazard ratio.

### Impact of obesity on the effects of atrial fibrillation ablation on functional symptom severity

In the overall population, AF ablation was associated with significantly lower odds of being in a higher NYHA functional class throughout follow-up compared to drug therapy (OR, 0.81; 95% CI, 0.68–0.95; *P* = 0.012). In continuous analysis, there was no significant interaction between BMI and the treatment effect (*P* for interaction = 0.997). Similarly, in categorical analysis using a threshold of BMI ≥ 30 kg/m^2^, both obese and non-obese patients had lower odds of being in a worse NYHA class after ablation (*P* for interaction = 0.702). However, across treatment groups, obese patients consistently had worse NYHA functional class than non-obese patients. *Figure 5* displays the model-predicted percentages of patients in NYHA class ≥ II following each intervention, pooled across all follow-up visits and stratified by obesity status. Regarding AF symptom severity, AF ablation resulted in significantly lower odds of being in a higher CCS class over the entire follow-up period (OR, 0.58; 95% CI, 0.52–0.65; *P* < 0.001). In continuous analysis, there was no significant interaction between BMI and treatment effect (*P* for interaction = 0.989). In categorical analysis, using a threshold of BMI ≥ 30 kg/m^2^, these significant lower odds were confirmed in the obese and non-obese patients, without treatment-effect modification being observed by obesity status (*P* for interaction = 0.980).

**Figure 5:**
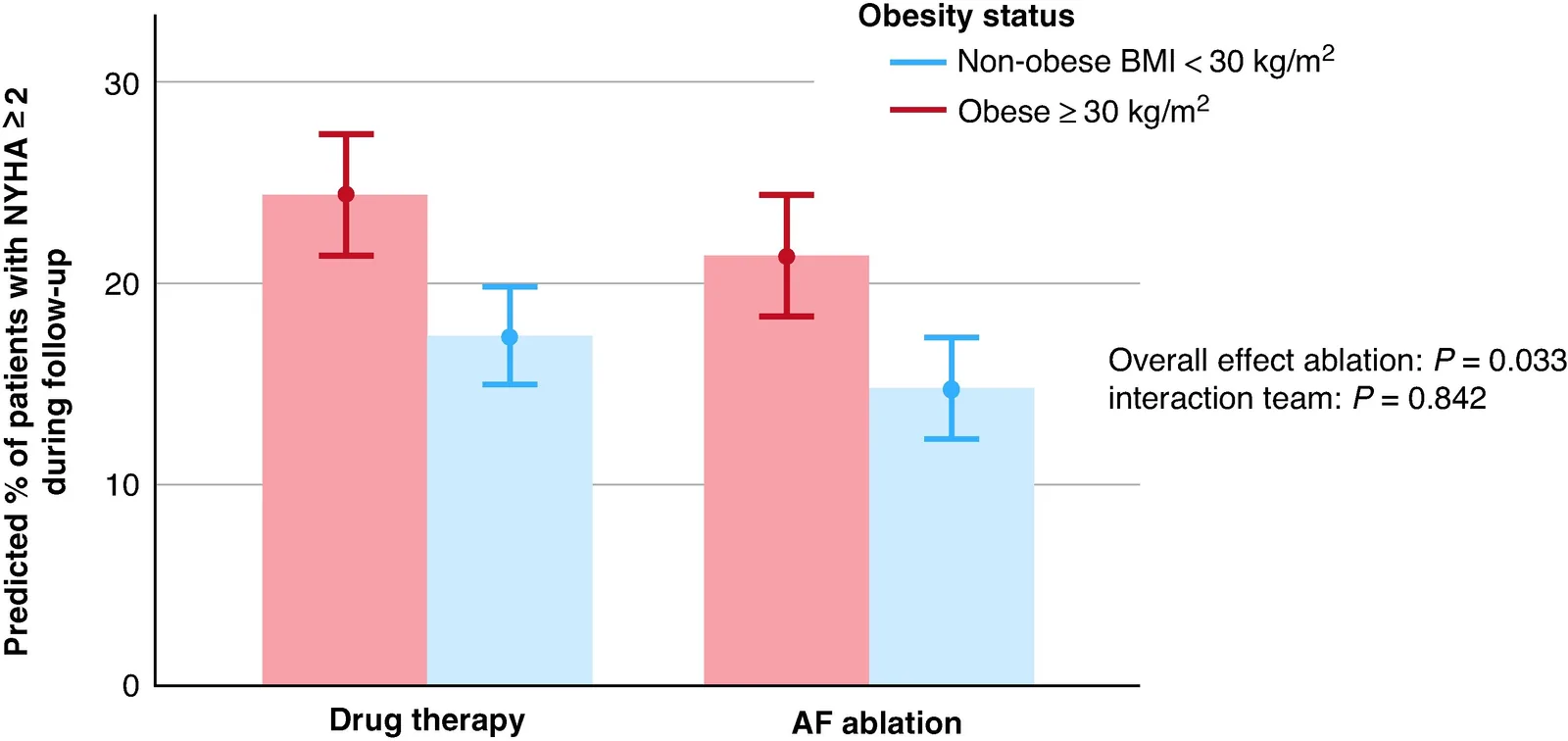
Generalized estimating equations model-predicted percentage of patients with NYHA class ≥ II during follow-up, stratified by obesity status (non-obese, BMI < 30 kg/m^2^; obese, BMI ≥ 30 kg/m^2^) and treatment assignment (AF ablation vs. drug therapy). Error bars represent 95% confidence intervals. AF, atrial fibrillation; BMI, body mass index; NYHA, New York Heart Association.

### Impact of obesity on the effects of atrial fibrillation ablation on atrial fibrillation recurrence

In the overall population, AF ablation was associated with a significantly lower odds of AF recurrence compared to drug therapy (OR, 0.37; 95% CI, 0.32–0.44; *P* < 0.001). In categorical analysis using a BMI threshold of ≥30 kg/m^2^, treatment benefit of ablation was confirmed in both obese and non-obese patients, without evidence of treatment-effect modification (*P* for interaction = 0.355). Similarly, when BMI was analysed as a continuous variable, there was no significant interaction observed between BMI and the treatment effect (*P* for interaction = 0.224).

## Discussion

In this *post hoc* analysis of the CABANA trial, we observed that baseline obesity was associated with distinct baseline characteristics and clinical outcomes following catheter ablation for AF. Our analysis revealed several important observations: (i) obesity is highly prevalent among patients undergoing AF ablation, affecting over half the studied population and representing a distinct clinical phenotype; (ii) higher BMI is associated with increased CV hospitalizations and functional incapacity; and (iii) while the relative treatment effects of ablation compared to drug therapy did not significantly differ across BMI categories (no clear treatment-effect modification), obese patients, particularly those with Class II obesity or higher, experienced worse outcomes, highlighting the need for a more comprehensive and multifactorial approach to treatment.

The high prevalence of obesity (51%) in this cohort reflects the growing burden of cardiometabolic disease in AF populations. Obese patients exhibited a distinct clinical phenotype. They were characterized by younger age, greater comorbidity burden (notably hypertension, diabetes, sleep apnoea, and coronary artery disease), and higher symptom severity. Conversely, non-obese patients more frequently had valvular heart disease, suggesting a different remodelling substrate.

Our analysis demonstrates a statistically significant association between BMI and the composite outcome of CV hospitalization or all-cause death in patients treated for AF, driven primarily by the heightened hospitalization risk in Class II obesity or higher. Importantly, CV hospitalization is a broad clinical endpoint and may reflect the greater cardiometabolic and comorbidity burden associated with obesity rather than an AF-specific or rhythm control-related effect. This is consistent with prior findings linking obesity to greater healthcare utilization and underscores the systemic impact of obesity-related cardiometabolic dysfunction.^12,13^ From a functional standpoint, obesity was associated with a worse NYHA class during follow-up. This functional limitation likely reflects the cumulative burden of excess body weight itself, co-existing conditions, and obesity-related atrial remodelling.^20,21^ From an electrical perspective, patients with obesity had more persistent and long-standing AF, indicating indeed more electromechanical remodelling of the left atrium. Furthermore, the worse functional status likely also reflects reduced cardiac reserve and underlying haemodynamic dysfunction common in obesity. Despite these elevated risk profiles in obese patients, our analysis did not identify a statistically significant interaction between obesity and the treatment effect of catheter ablation across multiple endpoints, including clinical outcomes, functional status, and AF recurrence. The absence of clear treatment-effect modification in interaction analyses suggests that the relative treatment effects of AF ablation are of similar magnitude in both obese and non-obese patients.

While the relative effects of AF ablation seemed to be maintained in obese patients, their residual risk for adverse outcomes and functional incapacity remained the highest. This indicates that a one-size-fits-all approach is inadequate. Ablation is a structural intervention, effective at isolating arrhythmogenic triggers, but it does not modify the underlying metabolic and inflammatory drivers of atrial remodelling that are particularly prominent in the distinct phenotype of obese AF patients. Importantly, previous studies have shown that lifestyle and weight loss interventions meaningfully improve exercise capacity and symptom burden in AF cohorts.^17,22^ These benefits are reinforced by recent randomized and observational trials, and supported by meta-analyses, showing that sustained weight loss enhances both physical functioning and quality of life.^15,16,18^ Functional gains are further amplified when weight reduction is combined with management of comorbidities. Notably, a recent CABANA subanalysis demonstrated that patients with a high probability of heart failure with preserved ejection fraction (HFpEF), who typically present with higher BMI and a greater comorbidity burden, derive more pronounced improvements in functional status following ablation.^23^ This likely reflects haemodynamic alleviation and improved heart failure status. These findings underscore that although ablation can relieve haemodynamic burden, sustained metabolic and weight-directed strategies remain essential to address the underlying drivers of atrial remodelling. Recently, pharmacologic therapies have entered this therapeutic space, with glucagon-like peptide-1 receptor agonists being promising adjuncts. While direct evidence on their effect in patients with AF is still limited, emerging data from obesity and HFpEF trials support their potential role in improving functional status and symptom burden in patients with obesity-related atrial remodelling.^24^ Further randomized trials are needed to clarify their specific benefit in AF patients. Collectively, these findings support a paradigm shift towards an integrative strategy in which rhythm control is complemented by upstream therapies that address the metabolic, inflammatory, and haemodynamic factors driving both arrhythmia and physical impairment. However, contemporary European data highlight an important implementation gap, with formal lifestyle and risk-factor management remaining underused in clinical practice; for example, an EHRA survey reported that only 23.5% of patients with AF and elevated BMI were referred for formal dietary advice, while recent patient-level data similarly identified gaps in integrated comorbidity management.^25,26^

In the present *post hoc* analysis, obesity was not significantly associated with AF recurrence, and no significant treatment-by-obesity interaction was observed. These findings differ from several observational studies and meta-analyses reporting higher recurrence rates among patients with obesity following AF ablation.^12–14^ These prior studies differed from the present CABANA analysis in study design, patient selection, AF phenotype distribution, and rhythm-monitoring strategies. In CABANA, repeat ablation was permitted during follow-up and may therefore have influenced subsequent recurrence patterns, while the decision to reintervene depended on several clinical and procedural factors. Moreover, there was a greater prevalence of persistent AF in the obese subgroup, which is also typically associated with higher recurrence rates.^27^ Importantly, the absence of a statistically significant association in the present analysis should not be interpreted as evidence that obesity has no influence on AF recurrence, particularly given the exploratory nature of the analysis and the limited statistical power for detecting treatment-effect modification. Furthermore, detailed longitudinal data on AF burden, as defined by contemporary consensus recommendations, were not available, limiting a more granular assessment of recurrence patterns according to obesity status.^28^

### Study limitations

This analysis has limitations inherent to *post hoc* analyses. In particular, in the present analysis BMI/obesity-related treatment-effect modification was not pre-specified in the original CABANA statistical analysis plan and therefore exploratory. The interaction analyses should be considered hypothesis generating. Moreover, interaction testing is intrinsically limited in statistical power, particularly within smaller obesity subgroups, raising the possibility of Type II error and underestimation of modest true differences in treatment effect across BMI strata. Although CABANA was a randomized trial, treatment was not stratified by BMI or obesity status, and residual confounding may exist (factors such as differences in fitness, diet, or other psychosocial aspects could have influenced both the likelihood of obesity and the outcomes). Our use of BMI, while practical and widely accepted, may not fully capture adiposity-related mechanisms, such as visceral fat distribution, ectopic fat deposition, inflammatory/metabolic activity, or comorbid phenotypes often linked to obesity. Accordingly, our findings should not be interpreted as implying that BMI fully represents obesity-related atrial remodelling or its potential interaction with treatment. Moreover, obesity is closely associated with atrial cardiomyopathy and adverse atrial remodelling.^29^ Detailed atrial dimensional or volumetric data were not available in the present dataset, precluding assessment of whether atrial enlargement or remodelling mediated or modified the association between obesity and AF recurrence. This is relevant because atrial size and remodelling are established determinants of AF recurrence after pulmonary vein isolation.^30^ Detailed longitudinal data on weight change and other CV risk factors during follow-up were limited and inconsistently reported, preventing robust incorporation of dynamic changes, an important factor that could mediate outcomes in the obese groups. Lastly, CABANA trial was conducted during an earlier era of AF ablation (with enrolment from 2009 to 2016), with substantial operator-dependent variability in ablation strategies and lesion sets. Contemporary AF ablation has evolved considerably, including more standardized procedural workflows and the emergence of newer energy sources, such as pulsed-field ablation.^31,32^ These limitations temper our conclusions and suggest that our findings should be interpreted as hypothesis generating and further prospective studies will be needed to confirm the observed relationships here.

## Conclusions

In this *post hoc* analysis of the CABANA trial, baseline obesity emerged as a highly prevalent and clinically relevant comorbidity in patients undergoing treatment for AF. Obese patients displayed a distinct, high-risk phenotype marked by greater comorbidity burden, worse functional status, and higher rates of CV hospitalization or death. While the relative treatment effect of catheter ablation vs. drug therapy did not seem to significantly differ across BMI categories, the absolute clinical and functional outcomes in obese patients remained worse, underscoring the limitations of rhythm control as a standalone intervention in this subgroup. Our findings highlight the importance of a more individualized and comprehensive approach to AF management in patients with obesity, integrating upstream therapies that target obesity-related atrial remodelling and systemic cardiometabolic dysfunction.

## Supplementary Material

euag293_Supplementary_Data

## Data Availability

The CABANA trial data was obtained through the publicly available National Heart, Lung, and Blood Institute BioLINCC data repository, and this analysis was approved by the Cleveland Clinic Institutional Review Board as an exempt study. The authors have full access to all study data, take responsibility for its integrity and the accuracy of the analyses, and may agree to provide aggregate data upon reasonable request and approval by the Institutional Review Board.
