# Supplementary material for: Impact of obesity on clinical outcomes and the treatment effect of catheter ablation in atrial fibrillation: insights from CABANA
Source: Europace. 2026 Oct 29;28(10):euag293. doi: 10.1093/europace/euag293 (PMC13717916; doi:10.1093/europace/euag293)
Supplement: Supplementary file 1 [file euag293_supplementary_data.docx]

**SUPPLEMENTAL MATERIALS**

**Table S1: Baseline characteristics of the patient population, stratified by obesity status.**

|  | **Overall**  (n = 2,170) | **Non-obese**  **(BMI <35 kg/m²)** (n = 1,663) | **Obese**  **(BMI ≥35 kg/m²)**  (n = 507) | **P value** |
| --- | --- | --- | --- | --- |
| Clinical characteristics | | | | |
| Age (years) | 67.0 (62.0 – 72.0) | 68.0 (63.0 – 72.0) | 65.0 (59.0 – 69.0) | **<0.001** |
| Male | 1370 (63.1) | 1,072 (64.5) | 298 (58.8) | 0.020 |
| BMI (kg/m²) | 30.1 (26.5 – 34.6) | 28.5 (25.8 – 31.3) | 38.6 (36.6 – 41.4) | **<0.001** |
| Systolic blood pressure (mmHg) | 129.0 (120.0 – 140.0) | 128.0 (120.0 – 140.0) | 130.0 (119.0 – 140.0) | **0.019** |
| Diastolic blood pressure (mmHg) | 80.0 (70.0 – 84.0) | 80.0 (70.0 – 84.0) | 80.0 (70.0 – 84.0) | 0.632 |
| LVEF (%) | 57.0 (50.0 -61.8) | 57.0 (50.0 – 62.0) | 56.5 (50.0 – 61.0) | 0.850 |
| AF type | | | | |
| Paroxysmal | 935 (43.1) | 723 (43.5) | 212 (41.8) | 0.509 |
| Persistent | 1023 (47.1) | 784 (47.1) | 239 (47.1) |  |
| Long-standing persistent | 212 (9.8) | 156 (9.4) | 56 (11.0) |  |
| NYHA functional class | | | | |
| ≤1 | 1,387 (63.4) | 1,082 (65.6) | 305 (60.2) | **0.004** |
| 2 | 586 (27.2) | 441 (26.8) | 145 (28.6) |  |
| 3 | 180 (8.4) | 123 (7.5) | 57 (11.2) |  |
| 4 | 2 (<0.1) | 2 (0.1) | 0 (0.0) |  |
| CCS AF severity score | | | | |
| 0 | 226 (10.4) | 181 (10.9) | 45 (8.9) | 0.081 |
| 1 | 336 (15.5) | 263 (15.8) | 73 (14.4) |  |
| 2 | 696 (32.1) | 544 (32.7) | 152 (30.0) |  |
| 3 | 771 (35.5) | 577 (34.7 | 194 (38.3) |  |
| 4 | 141 (6.5) | 98 (5.9) | 43 (8.5) |  |
| Comorbidities | | | | |
| Hypertension | 1,750 (80.6) | 1300 (78.2) | 450 (88.8) | **<0.001** |
| Diabetes | 549 (25.3) | 336 (20.2) | 507 (42.0) | **<0.001** |
| Sleep apnea | 505 (23.3) | 287 (13.2) | 218 (43.0) | **<0.001** |
| Coronary artery disease | 417 (19.2) | 307 (18.5) | 110 (21.7) | 0.106 |
| Prior stroke | 126 (5.8) | 102 (60.1) | 24 (4.7) | 0.238 |
| Thromboembolic events | 89 (4.1) | 66 (4.0) | 23 (4.5) | 0.573 |
| Valvular disease | 261 (12.0) | 212 (12.7) | 18.8 (9.7) | 0.062 |
| Family history of AF | 248 (11.5) | 183 (11.0) | 65 (12.9) | 0.256 |

Caption: Values are presented as n (%) or as median (25th–75th percentiles). Abbreviations: BMI, body mass index; AF, atrial fibrillation; LVEF, left ventricular ejection fraction; NYHA, New York Heart Association; CCS, Canadian Cardiovascular Society.

**Table S2: Baseline characteristics of patients with and without available BMI data.**

|  | BMI missing (n = 34) | BMI available (n = 2,170) | P value |
| --- | --- | --- | --- |
| Clinical characteristics | | | |
| Age (years) | 68.5 (64.5 – 72.3) | 67.0 (62.0 – 72.0) | 0.332 |
| Male | 15 (44.1) | 1370 (63.1) | **0.031** |
| Systolic blood pressure (mmHg) | 130.0 (115.8 – 145.5) | 130.0 (116.0 – 144.0) | 0.783 |
| Diastolic blood pressure (mmHg) | 80.0 (71.5 – 85.5) | 80.0 (70.0 – 84.0) | 0.878 |
| LVEF (%) | 55.5 (28.6 – 60.0) | 57.0 (50.0 – 61.8) | 0.333 |
| AF type | | | |
| Paroxysmal | 11 (33.3) | 935 (43.1) | 0.476 |
| Persistent | 19 (57.6) | 1,023 (47.1) |  |
| Long-standing persistent | 3 (9.1) | 212 (9.8) |  |
| NYHA functional class | | | |
| ≤1 | 21 (67.8) | 1,387 (64.4) | 0.554 |
| 2 | 6 (19.4) | 586 (27.2) |  |
| 3 | 4 (12.9) | 180 (8.4) |  |
| 4 | 0 (0.0) | 2 (0.1) |  |
| CCS AF severity score | | | |
| 0 | 8 (25.0) | 226 (10.4) | 0.087 |
| 1 | 3 (9.4) | 336 (15.5) |  |
| 2 | 7 (21.9) | 696 (32.1) |  |
| 3 | 12 (37.5) | 771 (35.5) |  |
| 4 | 2 (6.3) | 141 (6.5) |  |
| Comorbidities | | | |
| Hypertension | 26 (78.8) | 1,750 (80.6) | 0.824 |
| Diabetes | 12 (36.4) | 549 (25.3) | 0.160 |
| Sleep apnea | 3 (9.1) | 505 (23.3) | 0.060 |
| Coronary artery disease | 7 (21.2) | 417 (19.2) | 0.823 |
| Prior stroke | 0 (0.0) | 126 (5.8) | 0.257 |
| Tromboembolic events | 1 (3.0) | 89 (4.1) | 1.000 |
| Valvular disease | 4 (12.1) | 261 (12.0) | 1.000 |
| Family history of AF | 4 (12.8) | 252 (11.5) | 0.786 |

Values are presented as n (%) or as median (25th–75th percentiles).

AF, atrial fibrillation; BMI, body mass index; CCS, Canadian Cardiovascular Society; LVEF, left ventricular ejection fraction; NYHA, New York Heart Association.

**Table S3: Patients receiving atrial fibrillation ablation across different randomization groups, stratified by obesity status.**

| Catheter ablation (n = 1,086) | | |
| --- | --- | --- |
| Non-obese (BMI <30 kg/m²) (n = 540) | Obese (BMI ≥30 kg/m²) (n = 546) | P value |
| 486 (90.0) | 499 (91.4) | 0.465 |
| Drug therapy (n = 1,084) | | |
| Non-obese (BMI <30 kg/m²) (n = 523) | Obese (BMI ≥30 kg/m²) (= 561) | P value |
| 136 (26.0) | 161 (28.7) | 0.340 |

Values are presented as n (%).

BMI, body mass index.

**Table S4: Catheter ablation-related adverse events in patients randomized to ablation, stratified by obesity status.**

|  | Non-obese patients (BMI <30 kg/m²) receiving ablation (n = 486) | Obese Patients (BMI ≥30 kg/m²) receiving ablation (n = 499) | P value |
| --- | --- | --- | --- |
| Ablation catheter insertion | | | |
| Hematoma | 12 (2.5) | 11 (2.2) | 0.835 |
| Pseudo aneurysm | 8 (1.6) | 3 (0.6) | 0.139 |
| Atrial venous fistula | 3 (0.6) | 1 (0.2) | 0.368 |
| Pneumothorax | 0 (0.0) | 1 (0.0) | / |
| Sepsis | 1 (0.2) | 0 (0.0) | / |
| DVT | 0 (0.0) | 0 (0.0) | / |
| Pulmonary embolus | 0 (0.0) | 0 (0.0) | / |
| Catheter manipulation within heart | | | |
| Cardiac tamponnade | 3 (0.6) | 5 (1.0) | 0.726 |
| TIA | 2 (0.4) | 1 (0.2) | 0.620 |
| Coronary occlusion | 0 (0.0) | 0 (0.0) | / |
| Myocardial infarction | 0 (0.0) | 1 0.2) | / |
| Complete heart block | 0 (0.0) | 0 (0.0) | / |
| Valvular damage | 0 (0.0) | 0 (0.0) | / |
| Ablation-related events | | | |
| Pericarditis | 7 (1.4) | 4 (0.8) | 0.379 |
| Phrenic nerve injury | 0 (0.0) | 0 (0.0) | / |
| Pulmonary vein stenosis | 1 (0.2) | 1 (0.2) | 1.000 |
| Esophageal ulcer | 2 (0.4) | 3 (0.6) | 1.000 |
| Atrial esophageal ulcer | 0 (0.0) | 0 (0.0) | / |
| Heparin induced bleeding | 0 (0.0) | 0 (0.0) | / |

Values are presented as n (%).

BMI, body mass index; DVT, deep venous thrombosis; TIA, transient ischemic attack.

**Table S5: Effect of obesity status on clinical endpoints in patients with atrial fibrillation after multivariable adjustment (age, type of AF, comorbidities).**

| Continuous BMI | | | | | | | | |
| --- | --- | --- | --- | --- | --- | --- | --- | --- |
|  | Hazard ratio | | | 95% CI | | | P value | |
| Composite endpoint | 1.01 | | | 1.00 – 1.02 | | | **0.033** | |
| CV hospitalization | 1.01 | | | 1.00 – 1.02 | | | **0.038** | |
| All-cause death | 1.01 | | | 0.97 – 1.04 | | | 0.663 | |
| Categorical analysis - Cutoff BMI ≥30 kg/m² | | | | | | | | |
|  | | Non-obese (n = 1,063) | Obese  (n = 1,107) | | Hazard ratio | 95% CI | | P value |
| Composite endpoint | | 531 (50.0) | 601 (54.3) | | 1.06 | 0.94 – 1.21 | | 0.354 |
| CV hospitalization | | 505 (47.5) | 579 (52.3) | | 1.08 | 0.94 – 1.23 | | 0.272 |
| All-cause death | | 63 (5.9) | 57 (5.1) | | 0.81 | 0.55 – 1.20 | | 0.292 |
| Categorical analysis - Cutoff BMI ≥35 kg/m² | | | | | | | | |
|  | | Class 0-I Obesity  (n = 1,574) | Class II-III Obesity  (n = 476) | | Hazard ratio | 95% CI | | P value |
| Composite endpoint | | 856 (51.5) | 276 (54.4) | | 1.19 | 1.03 – 1.38 | | **0.020** |
| CV hospitalization | | 821 (49.4) | 263 (51.9) | | 1.18 | 1.02 – 1.37 | | **0.031** |
| All-cause death | | 89 (5.4) | 31 (6.1) | | 1.26 | 0.81 – 1.98 | | 0.314 |

Values are presented as n (%).

BMI, body mass index; CI, confidence interval; CV, cardiovascular; HR, hazard ratio.

**Table S6: Treatment effect of AF ablation on hard clinical endpoints, stratified by obesity status.**

|  |  | Event rate ablation arm (n = 1,086) | Event rate drug arm (n = 1,084) | HR | 95% CI | P value | P value for interaction |
| --- | --- | --- | --- | --- | --- | --- | --- |
| Composite endpoint (CV hospitalization and/or all-cause death) | | | | | | | |
| Overall | | 529 (48.7) | 603 (55.7) | 0.88 | 0.78 – 0.99 | **0.032** |  |
| BMI cutoff 30 kg/m² | BMI <30 kg/m² | 252 (46.7) | 279 (53.3) | 0.84 | 0.71 -1.00 | 0.050 | 0.494 |
|  | BMI ≥30 kg/m² | 277 (50.7) | 324 (57.8) | 0.92 | 0.79 -1.08 | 0.325 |  |
| BMI cutoff 35 kg/m² | BMI <35 kg/m² | 406 (47.5) | 450 (52.6) | 0.85 | 0.75 -0.97 | **0.019** | 0.268 |
|  | BMI ≥35 kg/m² | 123 (53.0) | 153 (55.6) | 1.00 | 0.79 -1.27 | 0.993 |  |
| CV hospitalization | | | | | | | |
| Overall | | 513 (47.2) | 571 (52.7) | 0.90 | 0.80 -1.02 | 0.099 |  |
| BMI cutoff 30 kg/m² | BMI <30 kg/m² | 244 (45.2) | 261 (49.9) | 0.88 | 0.74 -1.04 | 0.135 | 0.609 |
|  | BMI ≥30 kg/m² | 269 (49.3) | 310 (55.3) | 0.94 | 0.80 -1.11 | 0.453 |  |
| BMI cutoff 35 kg/m² | BMI <35 kg/m² | 395 (46.3) | 426 (52.7) | 0.88 | 0.77 -1.01 | 0.062 | 0.316 |
|  | BMI ≥35 kg/m² | 118 (50.9) | 145 (52.7) | 1.02 | 0.80 -1.30 | 0.885 |  |
| All-cause death | | | | | | | |
| Overall | | 55 (5.1) | 65 (6.0) | 0.83 | 0.580 -1.19 | 0.309 |  |
| BMI cutoff 30 kg/m² | BMI <30 kg/m² | 27 (5.0) | 36 (6.9) | 0.71 | 0.43 -1.17 | 0.180 | 0.376 |
|  | BMI ≥30 kg/m² | 28 (5.1) | 29 (5.2) | 0.99 | 0.59 -1.66 | 0.965 |  |
| BMI cutoff 35 kg/m² | BMI <35 kg/m² | 42 (4.9) | 47 (5.8) | 0.83 | 0.55 -1.26 | 0.376 | 0.949 |
|  | BMI ≥35 kg/m² | 13 (5.6) | 18 (6.5) | 0.86 | 0.42 -1.75 | 0.674 |  |

Values are presented as n (%).

AF, atrial fibrillation; BMI, body mass index; CI, confidence interval; CV, cardiovascular; HR, hazard ratio.
